# Retraction: Low-dose chest CT for diagnosing and assessing the extent of lung involvement of SARS-CoV-2 pneumonia using a semi quantitative score

**DOI:** 10.1371/journal.pone.0346849

**Published:** 2026-04-09

**Authors:** The PLOS One Editors

The *PLOS One* Editors retract this article [1,2] due to concerns about compliance with the PLOS Human Subjects Research policy.

Following the publication of the article and Expression of Concern [1,2], PLOS investigated concerns pertaining to the reported ethical approval and the article’s adherence to *PLOS One*’s research ethics policies.

Specifically, concerns were raised that the study involved human participants but the article did not report Comité de Protection des Personnes (CPP) ethics approval, and the ethics approval number N°:2020–0012 cited in [1] was also reported in [3] despite apparent differences in the reported study period and sample sizes.

A representative of the Aix-Marseille Université Ethics Committee stated that the study complied with applicable local legislation and ethical standards. They stated that the study did not involve any sampling but only included analysis of patients’ medical data, and therefore the study did not require ethics approval from a CPP according to French law. The representative provided a copy of the ethics approval document N°:2020–0012 for editorial review.

PLOS reviewed the documentation provided by the institution and concluded that the documents did not fully resolve the concerns. Specifically,

- The ethics approval (N°:2020–0012) was issued by the local institutional review board: Institut Hospitalo-Universitaire Méditéranée-Infection on April 1, 2020, for a study titled “*Evaluation des aspects scannographiques dans le diagnostic et l’évaluation de la gravité des infections à COVID-19*”. The article’s Materials and methods section reports that this study involved a single-center retrospective study conducted between March 13 – March 20, 2020, and lists refusal to participate in the protocol as an exclusion criterion. The exclusion criterion raises concerns that the subjects were recruited prospectively and the ethics approval was granted retrospectively, calling into question the article’s compliance with *PLOS One*’s policies for Human Subjects Research.
- The signatures on the copy of the ethics approval document provided by the representative of the Aix-Marseille Université Ethics Committee do not appear to match the signatures of the ethics approval document provided in S1 File of [1,2].
- PLOS identified extensive co-publications between members of the approving ethics committee that issued the document N°:2020–0012 and multiple of the article’s authors, calling into question the independence and objectivity of the ethics committee, the board’s findings that the study did not require CPP approval, and the ethics approval granted.

Regarding the exclusion criteria listing refusal to participate in the protocol, the representative stated that this statement reflected patients’ right to refuse the secondary use of their clinical data and does not imply prospective recruitment or interventional research.

Regarding the differences in signatures of the ethics approval documentation, the representative stated that this was the result of the extraordinary circumstances under which the ethics review took place during the early phase of the COVID-19 pandemic. They state that the ethics board’s meeting took place over videoconference and that the initial approval was signed by the board’s secretary immediately upon approval, and that the official approval document was signed by the president of the committee during a second administrative phase. The representative also stated that they do not consider co-publications between researchers and committee members as conflicts of interest under institutional governance rules. They provided email communications related to the ethics approval discussion for editorial review.

Upon review of the communications provided by the representative, PLOS noted that communications dated April 1, 2020, included a copy of an early version of the article [1]. The ethics approval N°:2020–0012 is also dated April 1, 2020, suggesting that the authors had already accessed, processed, and analyzed patients’ medical data prior to receiving ethics approval for a retrospective analysis of these data. The *PLOS One* policies for Human Subjects Research, which also applies to studies involving human data, states that studies involving human participants must obtain prospective ethics approval.

PLOS has not been able to secure input from an independent official confirming whether or not this study should have been subjected to CPP approval as per French legislation.

AJ agrees with the retraction. JCL, MM, PP, and PB did not agree with the retraction. DR responded but expressed neither agreement nor disagreement with the editorial decision. TL, PAB, MC, JF, AB, JYG, and PH either did not respond directly or could not be reached.
